# Altered Synaptic Transmission and Excitability of Cerebellar Nuclear Neurons in a Mouse Model of Duchenne Muscular Dystrophy

**DOI:** 10.3389/fncel.2022.926518

**Published:** 2022-07-05

**Authors:** Tabita Kreko-Pierce, Jason R. Pugh

**Affiliations:** ^1^Department of Cellular and Integrative Physiology, University of Texas Health Science Center at San Antonio, San Antonio, TX, United States; ^2^Center for Biomedical Neuroscience, University of Texas Health Science Center at San Antonio, San Antonio, TX, United States

**Keywords:** DMD, *mdx*, cerebellum, cerebellar nuclei, synaptic transmission, firing, Purkinje cell

## Abstract

Duchenne muscular dystrophy (DMD) is generally regarded as a muscle-wasting disease. However, human patients and animal models of DMD also frequently display non-progressive cognitive deficits and high comorbidity with neurodevelopmental disorders, suggesting impaired central processing. Previous studies have identified the cerebellar circuit, and aberrant inhibitory transmission in Purkinje cells, in particular, as a potential site of dysfunction in the central nervous system (CNS). In this work, we investigate potential dysfunction in the output of the cerebellum, downstream of Purkinje cell (PC) activity. We examined synaptic transmission and firing behavior of excitatory projection neurons of the cerebellar nuclei, the primary output of the cerebellar circuit, in juvenile wild-type and *mdx* mice, a common mouse model of DMD. Using immunolabeling and electrophysiology, we found a reduced number of PC synaptic contacts, but no change in postsynaptic GABA_A_ receptor expression or clustering in these cells. Furthermore, we found that the replenishment rate of synaptic vesicles in Purkinje terminals is reduced in *mdx* neurons, suggesting that dysfunction at these synapses may be primarily presynaptic. We also found changes in the excitability of cerebellar nuclear neurons. Specifically, we found greater spontaneous firing but reduced evoked firing from a hyperpolarized baseline in *mdx* neurons. Analysis of action potential waveforms revealed faster repolarization and greater after-hyperpolarization of evoked action potentials in *mdx* neurons, suggesting an increased voltage- or calcium- gated potassium current. We did not find evidence of dystrophin protein or messenger RNA (mRNA) expression in wild-type nuclear neurons, suggesting that the changes observed in these cells are likely due to the loss of dystrophin in presynaptic PCs. Together, these data suggest that the loss of dystrophin reduces the dynamic range of synaptic transmission and firing in cerebellar nuclear neurons, potentially disrupting the output of the cerebellar circuit to other brain regions and contributing to cognitive and neurodevelopmental deficits associated with DMD.

## Introduction

Duchenne muscular dystrophy (DMD), the most common form of childhood muscular dystrophy, is caused by mutations in the *DMD* gene, which encodes the protein dystrophin (Emery, 1991). Dystrophin is highly expressed in skeletal muscle where it is incorporated into the dystrophin-associated glycoprotein complex and links the actin cytoskeleton to the extracellular matrix. Loss of dystrophin destabilizes the cellular membrane of muscle cells during contraction, resulting in progressive muscle degeneration and weakness commonly associated with DMD (Ervasti and Campbell, 1993; Michele and Campbell, 2003; Allikian and McNally, 2007; Ervasti, 2007; Gao and McNally, 2015). Individuals with DMD also display a range of non-progressive cognitive deficits, including reduced IQ scores (Billard et al., 1992; Cotton et al., 2001, 2005; Ricotti et al., 2016) and impaired verbal reasoning (Karagan et al., 1980; Hinton et al., 2000), and show high comorbidity with neurodevelopmental disorders, such as autism spectrum disorder and attention deficit hyperactivity disorder (Wu et al., 2005; Hinton et al., 2009; Pane et al., 2012; Fujino et al., 2018; Darmahkasih et al., 2020). This suggests that dystrophin may be necessary for proper neuronal function and that disruption of specific brain circuits may contribute to cognitive aspects of DMD.

Dystrophin is expressed in at least five isoforms transcribed by unique internal promoters on the same gene. Isoforms are identified by size (Dp71 to Dp427) and have unique cellular distributions and functions. Full-length dystrophin (Dp427) is not only responsible for muscle degeneration in DMD, but also contributes to cognitive deficits (Taylor et al., 2010; Banihani et al., 2015) and neurodevelopmental disorders (Ricotti et al., 2016). Full length dystrophin protein expression has been observed in select regions of the central nervous system (CNS), including the hippocampus, cortex, amygdala, and cerebellum. The highest CNS levels of dystrophin expression in mice are found in cerebellar Purkinje cells (PCs; Lidov et al., 1993), suggesting cerebellar dysfunction may play a particularly important role in motor and non-motor phenotypes associated with DMD (Cyrulnik and Hinton, 2008). Previous studies have shown that dystrophin localizes to inhibitory postsynaptic densities in PCs (Knuesel et al., 1999; Briatore et al., 2020), and the loss of dystrophin severely reduces inhibitory synaptic input to these cells with little change in excitatory input. Reduced inhibition in PCs is due to both reduced clustering of postsynaptic GABA_A_ receptors (Knuesel et al., 1999; Grady et al., 2006) and reducing number of functional inhibitory synapses (Anderson et al., 2003; Kueh et al., 2008; Wu et al., 2022). *In vivo* studies have shown altered baseline (Stay et al., 2019) and stimulus-evoked firing (Wu et al., 2022) in PCs and impaired performance in motor tasks, such as reduced balance (Beastrom et al., 2011) and uncoordinated movement (Grady et al., 2006). These data suggest that the cerebellar circuit is significantly impaired by loss of dystrophin, possibly explaining, at least in part, the motor and cognitive deficits associated with DMD.

Previous studies examining changes in the cerebellar circuit due to loss of dystrophin expression have focused almost exclusively on synaptic transmission and firing in PCs. However, the output of the cerebellar circuit is primarily mediated by the activity of cerebellar nuclear (CbN) neurons. PC axons project to the CbN where they form extensive inhibitory synapses, which regulate synaptic plasticity (Aizenman et al., 1998; Pugh and Raman, 2006) and firing behavior (Gauck and Jaeger, 2000; Person and Raman, 2012) in CbN neurons. However, it is not currently known how loss of dystrophin might impair PC transmission or firing in these output neurons. To address this issue, we have examined synaptic transmission and action potential (AP) firing in glutamatergic projection neurons of the CbN in wild-type and *mdx* mice [*mdx* mice contain a non-sense point mutation in the DMD gene and lack global expression of dystrophin (Dp427)]. Using a combination of immunolabelling and whole-cell patch clamp electrophysiology, we found reduced PC synaptic input to CbN neurons. This was due to both reduced number of synapses and reduced replenishment rate of synaptic vesicles. Somewhat paradoxically, we found that spontaneous firing was increased in *mdx* CbN neurons, but evoked firing from a hyperpolarized baseline was reduced. Analysis of evoked AP waveforms revealed faster repolarization of the AP and larger after-hyperpolarization (AHP) in *mdx* neurons, consistent with the activation of a larger voltage- or calcium- activated K^+^ current in these cells. We did not find evidence of dystrophin mRNA or protein expression in CbN neurons, suggesting that the changes in synaptic transmission and firing in these cells are primarily due to changes in upstream PC firing (Wu et al., 2022). Together, these data suggest that transmission from PCs to CbN neurons is significantly reduced in *mdx* mice, particularly during high-frequency activity. Furthermore, firing properties of CbN neurons show a reduced dynamic range, due to both an increased baseline firing rate and reduced evoked firing. Reduced dynamic range in the output neurons may limit the ability of the cerebellar circuit to modify downstream activity, potentially contributing to the motor and cognitive deficits observed in DMD.

## Methods

### Animal Care and Use

All animal use and experimental procedures in these studies were approved by the Institutional Animal Care and Use Committee at the University of Texas Health at San Antonio. Mice were housed and cared for by the Laboratory Animal Resources at the University of Texas Health at San Antonio, which is fully accredited by the Association for Assessment and Accreditation of Laboratory Animal and licensed by the United States Department of Agriculture. Animals were kept at room temperature on a 12 h light/dark cycles with free access to water and food. The animals were housed socially with littermates.

### Immunohistochemistry

Deeply anesthetized mice were transcardially perfused with 1 × PBS and then in ice cold 4% paraformaldehyde in 1 × phosphate-buffered saline (PBS). Perfused brains were isolated and subsequently fixed overnight at 4°C in 4% paraformaldehyde. Parasagittal sections (50 μm thick) through the vermis and CbN of dissected cerebella were prepared using a vibratome (Leica Biosystems, Buffalo Grove, IL, USA) and subsequently blocked for 1 h at room temperature with 4% goat serum in PBS containing 0.1% Triton X-100. Sections were then incubated with primary antibody, mouse anti-dystrophin (1:20; MANDRA1, NovusBio, Centennial, CO, USA), mouse anti-calbindin (1:500, Sigma-Aldrich, St. Louis, MO, USA), chicken anti-GFP (1:1,000, ThermoFisher, Scientific, Waltham, MA, USA), and mouse anti-synaptotagmin 2 (1:500, znp-1, Zebrafish International Resource Center) at 4°C overnight. Sections were then incubated with secondary antibody for 2 h at room temperature. The secondary antibodies used in this study were goat-anti mouse conjugated to either Alexa 488, Alexa 567, or Alex 647 (1:400, Life Technologies, Carlsbad, CA, USA) and goat anti-chicken (H+L) antibody conjugated to Alexa 488 (1:400, ThermoFisher Scientific, Walthm, MA, USA). Sections were mounted on slides using FluoroShield mounting medium (Sigma-Aldrich, St. Louis, MO, USA). Images were acquired using a confocal microscope (LSM 710, Zeiss, Oberkocken, Germany). All post-image analysis was done using ImageJ (NIH) software. Dystrophin expression was measured by drawing a region of interest around the soma of PCs or CbN neurons. Background fluorescence, measured in the granule cell layer or white matter tracks, was subtracted from all fluorescent measures. To control for variation in labeling efficiency and to provide a positive control, we also measured dystrophin labeling around blood vessels in each image. The astrocytic end-feet surrounding the blood vessels primarily express shorter dystrophin isoforms which are detected by the MANDRA1 antibody but are not affected by the point mutation in *mdx* mice.

### Brain Slice Preparation

For these studies, *mdx* mice (Jackson labs 001801) were crossed with the Thy-1 YFP-16 line (Jackson Labs 003709), which has previously been shown to express YFP exclusively in glutamatergic projection neurons in the lateral and interposed cerebellar nuclei (Bagnall et al., 2007; Kodama et al., 2012). This allowed us to study the effects of dystrophin deficiency specifically in the glutamatergic output neurons of the CbN. We used male mice in all experiments because DMD is an x-linked gene and human dystrophinopathies are primarily observed in males. Acute parasagittal brain slices including the lateral and interposed nuclei were prepared from the cerebella of male *mdx*:Thy-1 YFP-16 mice and wild-type littermate (Thy-1 YFP-16) controls (P14-P23). Mice were deeply anesthetized with isoflurane before rapid dissection of the cerebellum. The cerebellum was immediately placed in ice-cold oxygenated (95% O_2_ and 5% CO_2_) artificial cerebrospinal fluid (aCSF) containing (in mM): 119 NaCl, 26.2 NaHCO_3_, 2.5 KCl, 1.0 NaH_2_PO_4_, 11 glucose, 2 CaCl_2_, 1.3 MgCl_2_. Slices (170–200 μm) were cut from the lateral hemispheres of the cerebellum using a vibratome (Leica Biosystems, Buffalo Grove, IL, USA) and transferred to a recovery chamber filled with oxygenated aCSF. Slices were incubated at 34°C for 30 min after which they were allowed to return to room temperature and kept under continuous oxygenation until transferred to the recording chamber.

### Electrophysiology

All electrophysiological recordings were made at near physiological temperature (32–34°C). During recordings, slices were superfused with warmed oxygenated aCSF at a flow rate of ~2 ml/min using a recirculating pump (Cole-Parmer, Vernon Hills, IL, USA). Cells in the CbN were visualized with Dodt contrast video microscopy using a 60× water-immersion objective on an upright SliceScope Pro microscope (Scientifica, East Sussez, UK). YFP+ CbN neurons were visually identified using a GFP filter and an epifuorescence LED light source (470 nm, CoolLED, Andover, UK). Patch pipettes were pulled from borosilicate glass (2–4 MΩ) on a Fleming/Brown micropipette puller (Sutter Instruments, Novato, CA, USA). GABA_A_ receptor-mediated currents were isolated by the addition of 10 μm NBQX and 10 μm CPP to the bath solution to block AMPA and NMDA receptors, respectively. In experiments measuring miniature inhibitory postsynaptic currents (mIPSC), recording pipettes were filled with an internal solution containing (in mM): 135 KCl, 10 HEPES, 2 Na-ATP, 0.2 Na-GTP, 2 MgCl_2_, and 0.1 EGTA (pH 7.3–7.4, 285–295 mosm). 1 μm TTX was added to the bath aCSF solution to block voltage-gated Na^+^ channels. In experiments measuring GABA-uncaging currents or evoked IPSCs, the pipettes were filled with an intracellular solution containing (in mM): 135 CsCl, 10 HEPES, 4 Na-ATP, 0.5 Na-GTP, 5 EGTA, and 2 QX-314 (pH 7.3–7.4, 285–295 mosm). During GABA uncaging experiments, 60 μm RuBi-GABA (Tocris, Bristol, UK) was included in the recirculating bath solution. GABA was uncaged by brief (5 ms) illumination from a 470 nm LED light source (CoolLED, Andover, UK) with a 30 s inter-sweep interval to allow for the clearance of GABA between sweeps. Uncaging experiments were performed in a darkened room to prevent unwanted uncaging of RuBi-GABA in the recirculating bath solution. For experiments measuring evoked IPSCs, synaptic currents were elicited by placing a patch pipette filled with aCSF in the CbN or surrounding white matter near (40–100 μm) the soma of the patched cell. The paired-pulse ratio (PPR) was measured by delivering a pair of stimuli with a 50 ms interstimulus interval (ISI). IPSCs were also measured during high-frequency trains of stimulation (40 stimuli at 100 Hz). Steady-state depression was measured by averaging the last 5 IPCS of the 100 Hz stimulation. The IPSC amplitudes were normalized to the amplitude of the first IPSC in order to account for potential differences in the number of axons/synapses activated. To estimate release probability, the size of the readily releasable pool (RRP), and vesicle replenishment rate, we stimulated trains of IPSCs and plotted the cumulative IPSC amplitudes against stimulus number (Train Method) (Schneggenburger et al., 1999; Thanawala and Regehr, 2013). In order to account for potential differences in the number of axons/synapses activated by the extracellular stimulation across cells, we normalized IPSC amplitudes to the value of the first IPSC before plotting the cumulative IPSC. We then fit the last 7 points of the curve (stimuli 33–40) with a linear function and extrapolated the *y*-intercept and slope of this fit for each cell. When plotting normalized IPSCs rather than raw IPSC values, the *y*-intercept represents the readily releasable pool normalized to the number of vesicles released during the first IPSC, which we term the relative releasable pool. The release probability was calculated by dividing the amplitude of the first IPSC by the *y*-intercept; in this case, the reciprocal of the *y*-intercept as the first IPSC amplitude is always 1. The slope of the linear fit is proportional to the vesicle replenishment rate. We also used a second, independent method to measure release probability and RRP (EQ Method; Elmqvist and Quastel, 1965). In this analysis, IPSCs were again normalized to the first IPSC amplitude and each IPSC was then plotted against the cumulative IPSC amplitude. A linear fit was made to the first 5 points. The relative RRP was derived from the *x*-intercept of the linear fit and the probability of release was calculated by dividing the *x*-intercept by the amplitude of the first IPSC.

To measure vesicle replenishment rate, we delivered a train of high-frequency stimulation (40 stimuli at 100 Hz) to deplete the vesicle RRP. The time course of IPSC recovery was measured by evoking a single test stimulus delivered after a recovery interval (ranging from 50 to 4,000 ms) to assess recovery of the vesicle pool. Recovery of the vesicle pool was calculated by the ratio of the recovery IPSC to the first IPSC in the train. Reported IPSC values and display traces were obtained from the average of at least 10 sweeps per cell.

Spontaneous firing in YFP+ CbN neurons was measured during cell-attached recordings without synaptic blockers in the bath solution or during whole-cell current clamp recordings (in *i* = 0 mode) with 10 μM NBQX, 10 μM CPP, and 100 μM picrotoxin (PTX) included in the bath solution to block fast synaptic transmission. To measure cell excitability, YFP+ CbN neurons were held in current clamp mode and a hyperpolarizing current was injected into the cells to maintain a baseline membrane potential of −60 mV. From this baseline, APs were evoked by depolarizing current injections (200 ms, 20–150 pA) through the patch pipette. Fast synaptic transmission was blocked in these experiments. AP waveform including threshold, amplitude, half-width, maximal rates of depolarization and repolarization, and AHP amplitude, were measured from the first AP evoked at each current injection level. Membrane resistance was determined from step hyperpolarizing current injections.

Access resistance was monitored throughout all recordings. Cells that did not maintain a stable access resistance were not included for further analysis. Whole-cell current- and voltage-clamp recordings were made using a Multiclamp 700 B amplifier (Molecular Devices, Sunnyvale, CA, USA), filtered at 5 kHz and digitized at 50 kHz. Data were collected using pCLAMP software (Axon Instruments) and analyzed with IGOR Pro software (Wavemetrics, Portland, OR) and MiniAnalysis software (Synaptosoft, Fort Lee, NJ).

### Computational Modeling of Synaptic Parameters

In order to explore how changes in synaptic parameters influence short-term depression and analysis of cumulative IPSCs, we built a simple computational model of synaptic transmission during repetitive activation. In this model, the postsynaptic response amplitude was determined by the following formula:


$$\begin{array}{l} \text{Postsynaptic response }\left(\text{pA}\right)={\text{N}}^{*}{\text{P}}_{\text{r}}^{*}\text{Q}. \end{array}$$

Where, “N” represents the number of releasable vesicles, P_r_ represents the probability of release, and Q represents the postsynaptic response following the release of a single vesicle. The model also includes a vesicle replenishment rate that is constant throughout the stimulus train. We used this model to simulate synaptic responses during trains of repetitive stimulation while systematically modifying the release probability or vesicle replenishment rate. Simulated responses were plotted to show short-term depression and cumulative IPSC analysis (using both the train and EQ methods).

### Single Cell Patch qRT-PCR

Cytoplasm containing mRNA was collected through a whole-cell patch clamp pipette by applying a light suction until the cell cytoplasm was aspirated (Citri et al., 2011; Cadwell et al., 2016). The patch pipette was gradually raised above the slice and the content of the pipette was collected into an RNase-free PCR tube containing a lysis buffer. The cells were immediately stored at −80°C until further processing for single-cell qRT-PCR. Single-cell gene expression analysis was performed in collaboration with the Bioanalytic and Single-cell core (BASiC) at UT Health at San Antonio. The BASiC core was supported by the Cancer Prevention Research Institute of Texas (RP150600) and the Office of the Vice President of Research of UT Health at San Antonio. Single-cell patch qRT-PCR was carried out as previously described (Chen et al., 2013). Briefly, single cells in lysis buffer were thawed, vortexed, and spun down. Contamination from genomic DNA was reduced by treating the cells with DNase I. Reverse transcription (RT), preamplification, and PCR amplification were carried out according to the protocol of single-cell gene expression. Target genes were amplified using BioMark system with 1X SsoFast Eva-Green supermix with low ROX (172–5211, Bio-Rad, Hercules, CA) and 1X DNA binding dye sample loading reagent (100–3738, Fluidigm). In each chip assay, universal mouse RNA (200 pg) from mouse normal tissues (R4334566-1, BioChain, Newark, CA) and no template control (NTC) served as positive and negative controls. Quantitative PCR products were detected using Fluidigm BioMark HD system according to the protocol: Gene expression with the FlexSix IFC using Delta Gene assays (100–7717 B1, Fluidigm). GE Flex Six Fast PCR+Melt v1 program was used to collect the cycle threshold (CT) values. Raw CT values were obtained from the Fluidigm Biomark software and inverted (25-CT) to generate a log2-based scale for gene expression analysis and presentation. All data were normalized to actin values for each cell. The following primer pairs were used to detect VGlut2 and dystrophin expression in the current study: VGlut2: Forward (5′-CTGAGAAGAAGGCTCCGCTAT-3′) Reverse (5′-ATGCCGAAGGATATGCAGAAG-3′), Dp427: Forward (5′-GGAAAGCAACACATAGACAACCT-3′) Reverse (5′-GGGCATGAACTCTTGTAGATCC-3′), dystrophin C-terminus: Forward (5′-CGAGACCCAAACCACTTGTTG-3′), Reverse (5′-GGTCAGCTAAAGACTGGTAGAGC-3′).

Dystrophin mRNA expression was measured in YFP+ CbN neurons from wild-type mice. We also included cytoplasmic material from wild-type PCs and granule cells as positive and negative controls, respectively. Since the *mdx* mutation is a point mutation and not a full knockout of the DMD gene, we did not compare mRNA data between wild-type and *mdx* cells.

### Data Analysis

Data was analyzed in IgorPro (Wavemetrics, Lake Oswego, OR) using the Neuromatic toolkit (Rothman and Silver, 2018) and custom macros. Statistical significance was determined using two-tailed unpaired Student's *t*-tests in Excel (Microsoft, Redmond, WA). Statistical values of *P* ≤ 0.05 were considered significant.

## Results

The cerebellar nuclei contain multiple types of neurons, including at least two classes of projection neurons, GABAergic neurons that project directly to the inferior olive and glutamatergic neurons that project widely throughout the CNS, including the ventral tegmental area and multiple regions of the neocortex and hippocampus (*via* the thalamus). In order to understand how the loss of dystrophin (Dp427) disrupts the output of the cerebellum, we investigated changes in PC synaptic transmission and firing in glutamatergic projection neurons of the CbN. These neurons were identified by crossing the *mdx* mouse line into the Thy1-YFP16 line (Feng et al., 2000), a line which selectively expresses YFP in glutamatergic neurons in the lateral and interposed nuclei of the CbN (Bagnall et al., 2007; Kodama et al., 2012). Using single-cell qRT-PCR, we confirmed that YFP+ neurons in the CbN express VGlut2, a marker of glutamatergic neurons in the CbN (Supplementary Figure 1). Thus, for current studies, we utilized Thy1-YFP16 mouse line and performed all the analysis on YFP+ neurons in the CbN.

### Loss of Long-Form Dystrophin (Dp427) Disrupts the Structural and Functional Properties of PC-CbN Synapses

**Figure 1:**
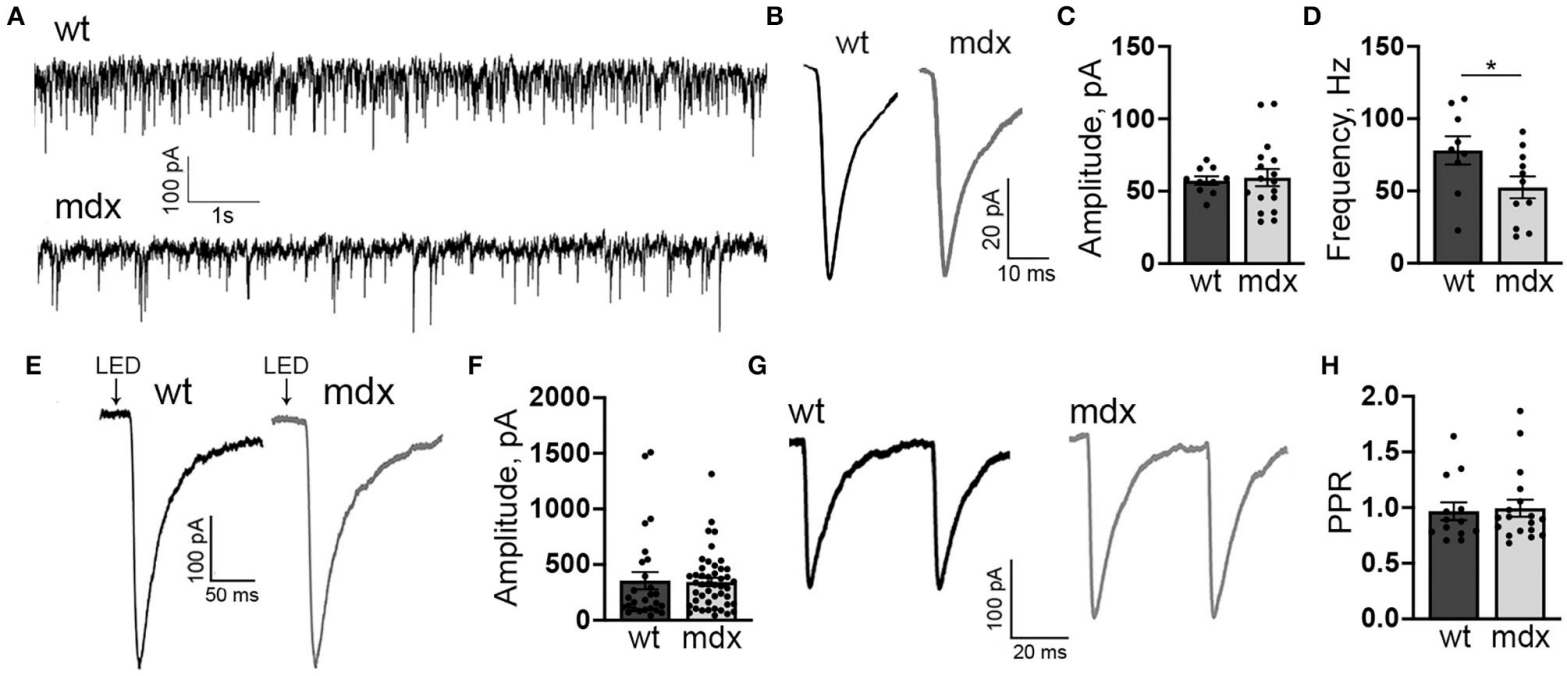
*mdx* CbN neurons show reduced mIPSC frequency. **(A)** Representative trace of mIPSC recording in YFP+ CbN neurons from wild type (top trace) and *mdx* (bottom trace) mice. Scale bar: 100 pA, 1 s. **(B)** Representative average mIPSC traces from a wild-type (left, black trace) and *mdx* (right, gray trace) CbN neuron. Traces are averages of all mIPSCs over a 2 min recording. Scale bar: 20 pA, 10 ms. **(C,D)** Summary graphs of mean mIPSC amplitude **(C)** and frequency **(D)** from wild-type (dark gray bars, *n* = 9 cells from 3 animals) and *mdx* (light gray bars, *n* = 11 cells from 4 animals) CbN neurons. **(E)** Representative current traces following photolytic uncaging of RuBi-GABA recorded from YFP+ CbN neurons of wild-type (left, black trace) and *mdx* (right, gray trace) mice. Traces are averages of 6–10 sweeps. Scale bar: 100 pA, 50 ms. **(F)** Summary graph of mean GABA_A_ receptor current amplitude from wild-type (dark gray bars, *n* = 27 cells, 4 animals) and *mdx* (light gray bars, *n* = 46 cells, 5 animals) CbN neurons. **(G,H)** Representative traces of pairs of IPSCs (50 ms inter-stimulus interval) evoked in YFP+ CbN neurons **(G)** and a summary graph of the paired-pulse ratio **(H)** from wild-type (dark gray bar, *n* = 13 cells, 3 animals) and *mdx* (light gray bar, *n* = 18 cells, 4 animals) cells. Scale bar: 100 pA, 20 ms. Traces are averages of 6–10 trials. Error bars indicate SEM. ^*^p ≤ 0.05.

In order to measure changes in synaptic transmission due to loss of dystrophin at PC-CbN synapses, we made whole-cell patch clamp recordings from YFP+ neurons in the CbN of acute cerebellar slices from *mdx* and wild-type mice. We first measured miniature inhibitory synaptic currents (mIPSCs) in the presence of 1 μm TTX, 10 μm NBQX, and 10 μm CPP to block voltage-gated Na^+^ channels, AMPA receptors, and NMDA receptors, respectively. We did not find any difference in the mean amplitude (WT: 57.4 ± 2.9, *n* = 10; *mdx*: 59.3 ± 5.9 pA, *n* = 17, *p* = 0.81) or kinetics of mIPSCs between wild-type and *mdx* cells (Figures 1A–C; Table 1), suggesting that the expression or clustering of postsynaptic GABA_A_ receptors is not impaired in *mdx* CbN neurons, contrary to observations in PCs (Knuesel et al., 1999; Grady et al., 2006). This was further confirmed by recording GABA_A_ receptor-mediated currents in YFP+ CbN neurons evoked by photolytic uncaging of RuBi-GABA (60 μM) from a brief (5 ms) LED light flash (Figure 1E). We did not observe any difference in the photo-current amplitude (WT: 355.2 ± 77.7, *n* = 27; *mdx*: 344.1 ± 37.1 pA, *n* = 46, *p* = 0.88, Figures 1E,F) or kinetics (Table 1) between wild-type and *mdx* neurons, suggesting that total GABA_A_ receptor surface expression does not differ between genotypes.

**Table 1:** Kinetic properties of mIPSC and GABA-evoked currents.

	**Wild-type**	***mdx***	***p-*value**
**mIPSC [*****n*** **=** **10 (wt), 17 (*****mdx*****)]**			
Time to peak (ms)	2.4 ± 0.10	2.38 ± 0.14	0.91
10–90 rise time (ms)	1.54 ± 0.12	1.92 ± 0.20	0.18
τ_fast_ (ms)	6.25 ± 2.21	4.26 ± 1.12	0.38
τ_slow_ (ms)	13.4 ± 1.59	13.5 ± 1.43	0.95
**GABA current [*****n*** **=** **27 (wt), 46 (*****mdx*****)]**			
Time to peak (ms)	14.1 ± 0.46	14.8 ± 0.72	0.46
10–90 rise time (ms)	7.29 ± 0.38	7.21 ± 0.29	0.87
τ_fast_ (ms)	28.8 ± 3.8	31.6 ± 4.2	0.65
τ_slow_ (ms)	136.1 ± 15.1	130.8 ± 13.8	0.81

*Data presented as mean ± SEM with Student's t-test*.

The mIPSC frequency was generally quite high in CbN neurons (~80 Hz; Figure 1A) consistent with extensive PC innervation of these cells (Ito et al., 1964; Mouginot and Gähwiler, 1995; Gauck and Jaeger, 2000, 2003; Telgkamp and Raman, 2002; Pedroarena and Schwarz, 2003). However, we found that the mIPSC frequency was significantly lower in *mdx* CbN neurons (52.5 ± 7.3 Hz, *n* = 17) compared to wild-type neurons (78.1 ± 9.3 Hz, *n* = 10, *p* = 0.049, Figure 1D), suggesting that inhibitory synaptic transmission in the CbN may be impaired. The reduced mIPSC frequency could be due to a decrease in the release probability at PC-CbN synapses, or due to the reduced number of functional connections. In order to distinguish between these possibilities, we first measured the PPR (50 ms ISI) of evoked IPSCs in CbN neurons, a common measure of synaptic release probability. We found that evoked IPSCs displayed a PPR near one, consistent with earlier reports (Telgkamp and Raman, 2002), but the PPR was not different across genotypes (0.97 ± 0.08 vs. 0.99 ± 0.08, *n* = 13 and 18, *p* = 0.82, Figures 1G,H). This suggests that release probability at PC synapses is not reduced in *mdx* mice and raises the possibility that PC innervation of CbN neurons is altered.

**Figure 2:**
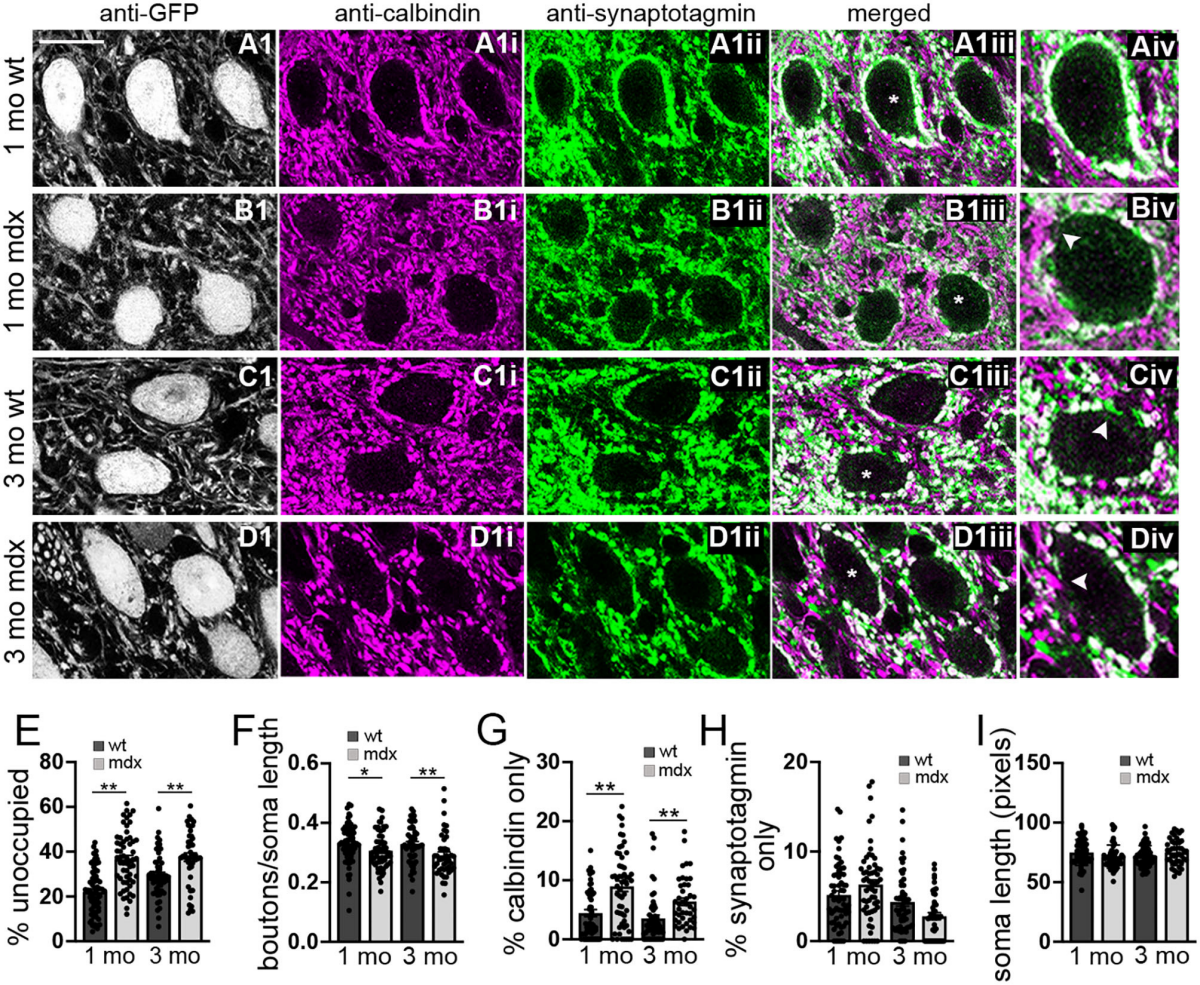
The number of anatomically defined PC boutons contacting CbN neurons is reduced in *mdx* mice. **(A–D)** Representative 40x confocal sagittal cerebellar slice images of YFP+ CbN neurons **(A1–D1)** and PC-CbN synapses from 1- and 3-month-old wild-type **(A,C)** and *mdx* **(B,D)** mice. Sections were immunolabelled for GFP (grayscale channel, labeling glutamatergic neurons, **A1–D1**), calbindin (magenta channel, PC marker, **A1i–D1i**) and synaptotagmin (green channel, presynaptic marker, **A1ii–D1ii**). Merged images of calbindin and synaptotagmin labeling are also shown **(A1iii–D1iii)**. **(Aiv–Div)** Enlarged CbN somata (indicated by asterisk (*) in **A1iii–D1iii**) showing the area around the CbN soma lacking PC boutons (arrowhead in **B1iv**), area occupied with PC axons (calbindin—only staining, arrowhead in **D1iv**) and area occupied by non-PC boutons (synaptotagmin—only staining, arrowhead in **C1iv**). Scale bar: 20 μm. **(E–H)** Summary graphs of mean values for length of CbN soma lacking PC boutons (**E**, % unoccupied), number of PC boutons surrounding each soma (**F**, boutons/soma length), and length of soma contacted by structures labeled by either calbindin only **(G)** or synaptotagmin only **(H)**. Data from 1- or 3-month-old wild-type [dark gray bars, *n* (1 mo) = 63, *n* (3 mo) = 53 cells] and *mdx* [light gray bars, *n* (1 mo) = 58, *n* (3 mo) = 43 cells] mice are shown. Three mice were analyzed per age and genotype. All values are normalized to the soma circumference of each cell. The circumference of YFP+ somata were not different between wild-type and *mdx* cells at either age **(I)**. Error bars indicate SEM. ^*^p ≤ 0.05, ^**^p ≤ 0.01.

To investigate this possibility, we used immunohistochemistry to label PC synaptic terminals around the soma of YFP+ CbN neurons (Figures 2A–D), taking advantage of the fact that PC terminals in the CbN are large (Telgkamp et al., 2004) and easily visualized in confocal images. PC terminals in the CbN were identified by co-labeling for calbindin (a PC marker) and synaptotagmin (a presynaptic marker). We found that the soma of wild-type CbN neurons are almost completely surrounded by PC terminals, with little or no membrane area unoccupied by PC terminals (1 mo: 22.1 ± 1.3% unoccupied, *n* = 63; 3 mo: 29.4 ± 1.4% unoccupied, *n* = 52; Figures 2Aiii,Aiv,C1iii,C1iv,E). However, PC innervation of CbN neurons from *mdx* mice was markedly reduced, measured by an increase in the percentage of the soma membrane unoccupied by PC terminals (1 mo: 36.7 ± 1.6% unoccupied, *n* = 58, *p* < 0.0001; 3 mo: 37.5 ± 1.9% unoccupied, *n* = 43, *p* = 0.00065, Figure 2Biv, arrow; Figure 2E), as well as a reduced density of boutons (1 mo: WT: 0.33 ± 0.008 boutons/μm, *n* = 63; *mdx*: 0.31 ± 0.008 boutons/μm, *n* = 58, *p* = 0.039; 3 mo: WT: 0.33 ± 0.009 boutons/μm, *n* = 52; *mdx*: 0.29 ± 0.01 boutons/μm, *n* = 43, *p* = 0.008, Figure 2F). Further analysis revealed a greater prevalence of PC axons (calbindin-only staining; Figure 2Div, arrow) adjacent to the soma of CbN neurons in *mdx* mice (1 mo: *p* = 0.00003, 3 mo: *p* = 0.001; Figure 2G), suggesting PC projections are present, but fail to form synaptic contacts with the soma. Innervation of the soma by synaptic terminals arising from neurons other than PCs (synaptotagmin-only staining; Figure 2Civ, arrow) remained unchanged (1 mo: *p* = 0.10, 3 mo: *p* = 0.06; Figure 2H), suggesting other synaptic contacts at the soma are minimally affected. Soma circumference was not different across genotypes (Figure 2I). These data suggest that PC innervation of glutamatergic CbN neurons is impaired by the loss of dystrophin, likely accounting for the reduction in mIPSC frequency observed in *mdx* neurons. This result is similar to the reduced inhibitory synapse number and mIPSC frequency found previously in PCs of *mdx* mice (Wu et al., 2022).

### The Vesicle Replenishment Rate Is Reduced in PC-CbN Synapses From *mdx* Mice

**Figure 3:**
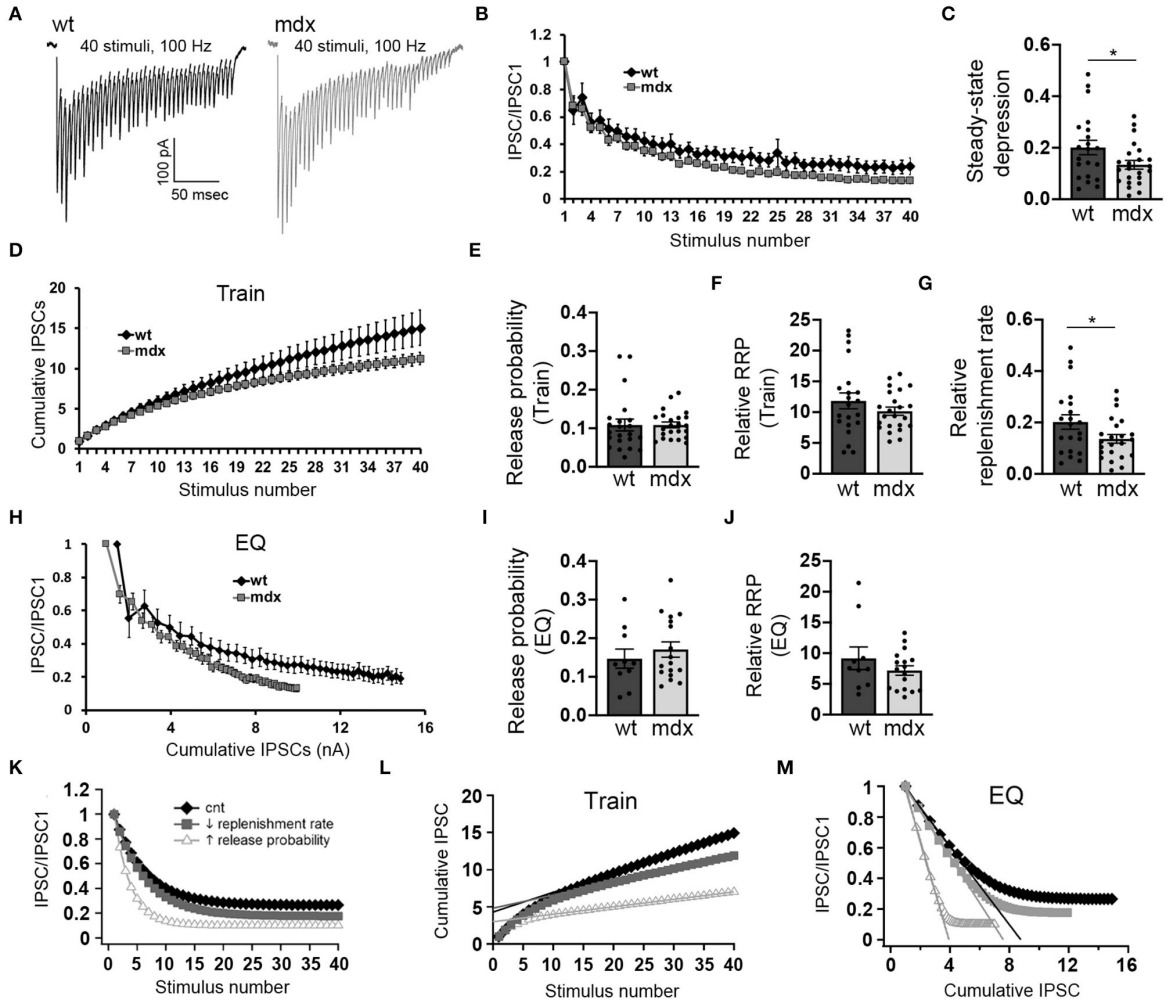
PC inputs to CbN neurons show greater short-term synaptic depression and slower recovery in *mdx* mice. **(A)** Representative traces of IPSCs evoked by 40 stimuli at 100 Hz recorded in wild-type (black trace) and *mdx* (gray trace) YFP+ CbN neurons. Scale bar: 100 pA, 50 ms. **(B)** Summary graph of normalized mean IPSC amplitudes as a function of stimulus number from wild-type (black diamonds, *n* = 10 cells, 4 animals) and *mdx* (gray squares, *n* = 17 cells, 5 animals) CbN neurons. **(C)** Summary graph of normalized steady-state IPSCs (mean of last 5 IPSCs) during 100 Hz stimulation. **(D)** Summary graph of cumulative IPSC amplitude (normalized to first IPSC) plotted as a function of stimulus number from wild-type (black diamonds, *n* = 17 cells, 5 animals) and *mdx* (gray squares, *n* = 17 cells, 5 animals) cells. **(E–G)** Graphs represent mean values for release probability **(E)**, relative RRP size **(F)**, and relative replenishment rate **(G)** calculated from cumulative IPSC plots (**D**, Train method). **(H)** Summary graph of normalized mean IPSC amplitudes plotted against cumulative IPSC amplitude (normalized to first IPSC) from wild-type (black diamonds, *n* = 10 cells, 4 animals) and *mdx* (gray squares, *n* = 17 cells, 5 animals) CbN neurons. Summary graph of release probability **(I)** and relative RRP size **(J)** calculated from cumulative IPSC plot (**H**, EQ method). **(K–M)** Graphs showing simulated synaptic responses from a simple computer model of vesicle release using control parameters (black diamonds), reduced vesicle replenishment rate (gray squares), or increased release probability (open triangles). For direct comparison with experimental data, graphs show simulated IPSC amplitudes and cumulative IPSC amplitudes (train method) plotted against stimulus number **(K,L)** and IPSC amplitudes plotted against cumulative IPSC amplitude (**M**; EQ method). Error bars indicate SEM. *p ≤ 0.05.

Purkinje cells often exhibit sustained high-frequency firing *in vivo* (Goossens et al., 2004; Shin et al., 2007; Cheron et al., 2009; Chaumont et al., 2013; Witter et al., 2013; Zhou et al., 2014). We therefore also assessed how loss of dystrophin affects high-frequency transmission at PC-CbN synapses. To do this, we made whole-cell recordings from YFP+ CbN neurons and stimulated high-frequency trains (40 stimuli at 100 Hz) of inhibitory input in acute slices from wild-type and *mdx* mice (Figure 3A). We found that the steady-state depression of IPSCs (average amplitude of the last 5 IPSC normalized to the first IPSC) is greater in *mdx* neurons compared to wild-type controls (WT 0.2 ± 0.13 *n* = 21; *mdx*: 0.13 ± 0.08, *n* = 23 *p* = 0.042; Figures 3B,C), suggesting that transmission at PC-CbN synapses may be particularly impaired during high-frequency activity. Increased steady-state depression could be due to increased release probability or reduced vesicle replenishment. To distinguish between these two likely mechanisms, we initially measured the vesicle release probability and size of the RRP (relative to the first IPSC) at PC-CbN synapses using two established methods, the “Train” method (Schneggenburger et al., 1999; Figure 3D) and the “EQ” method (Elmqvist and Quastel, 1965; Figure 3H), to analyze cumulative IPSCs (refer to Methods). Using both methods, we did not find a difference in the release probability [P_r_ (Train): WT: 0.11 ± 0.07, *n* = 22; *mdx*: 0.11 ± 0.04, *n* = 23, *p* = 0.99, P_r_ (EQ): WT: 0.15 ± 0.08, *n* = 10; *mdx* 0.17 ± 0.08, *n* = 17, *p* = 0.46; Figures 3E,I] or RRP [RRP (Train): WT 11.8 ± 1.3, *n* = 21; *mdx* 10.2 ± 0.67, *n* = 23, *p* = 0.24, RRP (EQ): WT 9.2 ± 1.85, *n* = 21; mdx 7.18 ± 0.77, *n* = 23, *p* = 0.26, Figures 3F,J] at PC-CbN synapses across genotypes, consistent with the lack of change in PPR (Figure 1H). Our data suggest that the increased steady-state depression observed during high-frequency stimulation is not due to increased probability of release or changes in RRP. Next, we tested the alternative possibility; that the increased steady-state depression is a result of reduced vesicle replenishment rate. We initially measured the slope of the linear fit (from Figure 3D) and found that it was significantly reduced in *mdx* neurons compared to wild-type controls (WT: 0.20 ± 0.13 *n* = 21; *mdx*: 0.13 ± 0.08, *n* = 23 *p* = 0.045, Figure 3G), suggesting a decrease in the vesicle replenishment rate. For comparison, we created a simple computational model of vesicle release and replenishment (refer to Methods). In this model, changes in synaptic responses observed in *mdx* mice (reduced steady-state depression and slope of the cumulative IPSC plot) were closely replicated by reducing the vesicle replenishment rate, but not by an increase in the release probability (Figures 3K–M).

**Figure 4:**
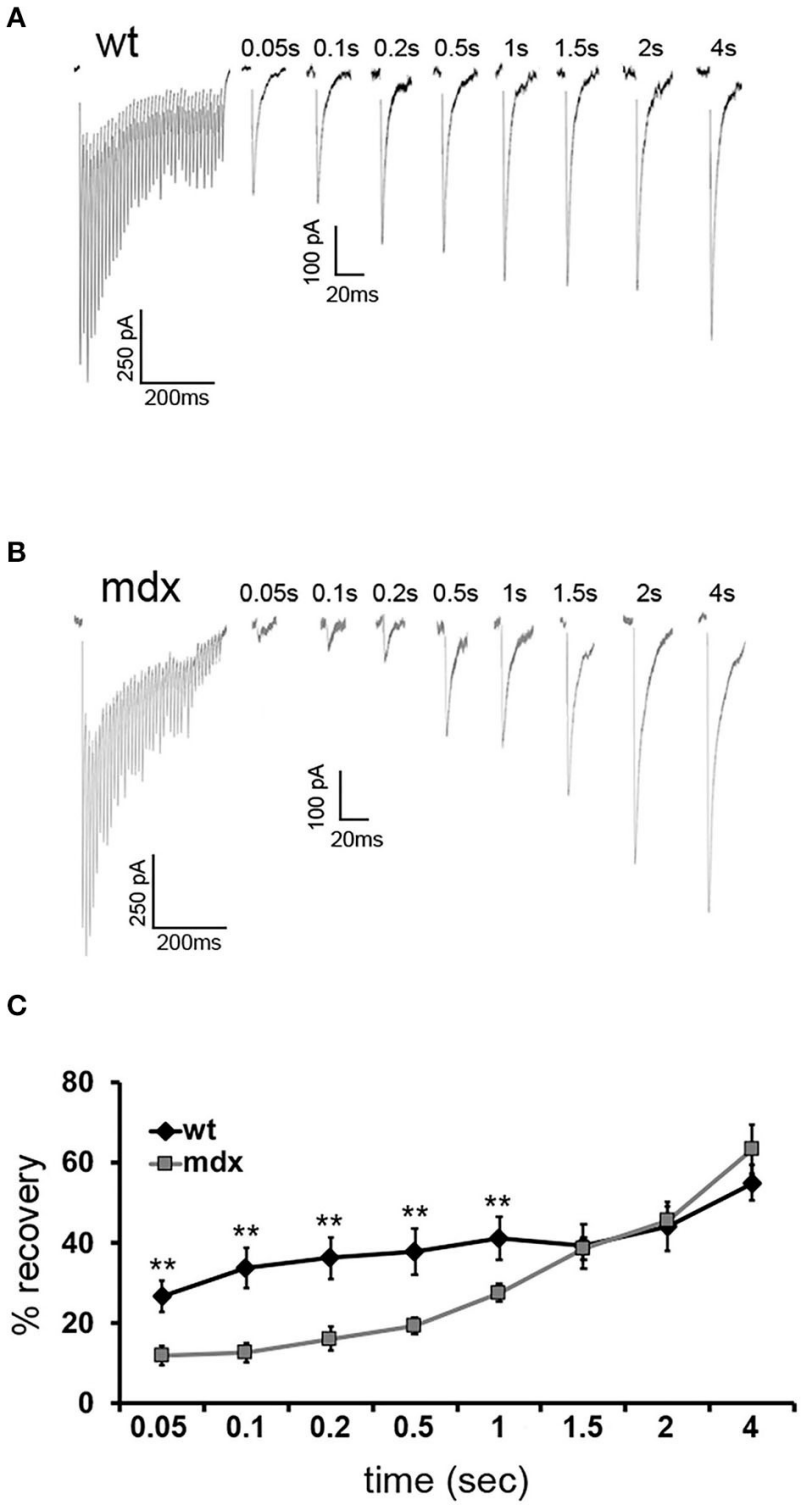
Recovery from short-term depression is slower in *mdx* CbN neurons. Representative traces of IPSCs evoked by a 100-Hz stimulus train followed by single IPSCs after a delay of 0.05–4 s from wild-type **(A)** and *mdx* **(B)** CbN neurons. Scale bar (100 Hz stimulation): 250 pA, 200 ms. Scale bar (IPSC recovery): 100 pA, 20 ms. **(C)** Summary graph of normalized mean recovery of IPSC amplitudes following 100 Hz stimulation from wild-type (black diamonds, *n* = 10 cells, 2 animals) and *mdx* (gray squares, *n* = 18 cells, 4 animals) cells. Traces are averages of 6–10 trials. Error bars indicate SEM. **p ≤ 0.01.

To measure the replenishment rate more directly, we depleted the vesicle RRP at PC-CbN synapses with trains of 40 stimuli at 100 Hz and then applied test stimuli at different time intervals following the end of the train (50–4,000 ms, Figures 4A,B). This analysis revealed that recovery from depression is reduced at PC-CbN synapses of *mdx* mice (Figure 4C, gray squares) immediately following 100 Hz stimulation. However, recovery from depression was not different between wild-type and *mdx* at time-points beyond 1.5 s, suggesting that fast vesicle replenishment may be specifically impaired at *mdx* PC-CbN synapse. These data suggest that loss of dystrophin may particularly impair high-frequency synaptic transmission at PC-CbN synapses due to reduced vesicle replenishment.

### Expression of Dystrophin in the Cerebellar Cortex and Nuclei

**Figure 5:**
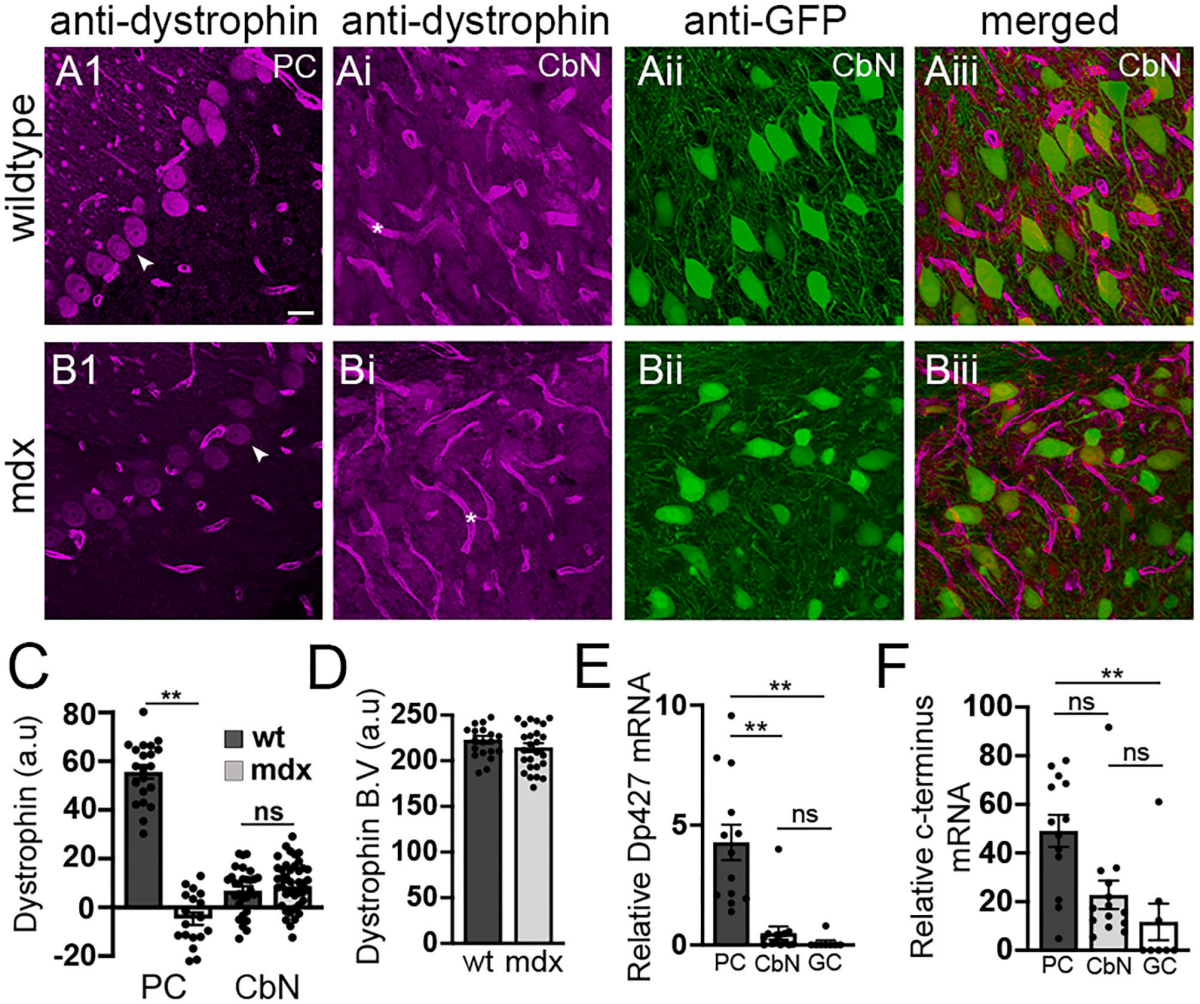
Dystrophin Dp427 isoform is not expressed in glutamatergic projection neurons of the CbN. **(A,B)** Representative 40x confocal sagittal cerebellar slice images of PC layer **(A1,B1)** and CbN **(Ai,Bi–Aiii,Biii)** from wild-type **(A)** and *mdx* mice **(B)** immunolabelled for dystrophin (magenta channel) and GFP (green channel, indicating glutamatergic projection neurons in CbN). **(Aiii,Biii)** are composite images of dystrophin **(Ai,Bi)** and GFP **(Aii,Bii)** labeling in the CbN. Arrowheads indicate PC somata **(A1,B1)**. Asterix (*) indicates blood vessels **(Ai,Bi)**. Scale bar: 20 μm. **(C)** Summary graph of mean intensity of dystrophin labeling (background subtracted) of PCs and YFP+ CbN neurons in wild-type [dark gray bars, *n* = 21 (PC) and 30 (CbN) cells from 2 animals] and *mdx* [light gray bars, *n* = 18 (PC) and 41 (CbN) cells from 2 animals]. **(D)** Graph of normalized mean intensity of dystrophin labeling of blood vessels in the CbN from wild-type (dark gray bars, *n* = 21 BV from 2 animals) and *mdx* (light gray bars, *n* = 25 BV from 2 animals) mice. **(E,F)** Summary graphs of mean mRNA expression (normalized to actin) of the Dp247 dystrophin isoform (measured using primers targeted to the N-teminus of dystrophin); **(E)**, and all dystrophin isoforms (measured using primers targeted to the C-teminus of dystrophin); **(F)** in PCs (dark gray bars, *n* = 13 cells from 2 animals), YFP+ CbN neurons (Cbn, light gray bars, *n* = 14 cells from 2 animals) and cerebellar granule cells (GC, white bars, *n* = 8 cells from 2 animals) using single cell patch qRT-PCR. Error bars indicate SEM. **p ≤ 0.01. ns, not significant.

Our data reveal that loss of long-form dystrophin impairs several key synaptic processes at the PC-CbN synapse. However, these impairments could be due to lack of dystrophin in the presynaptic PC and/or the lack of dystrophin from postsynaptic CbN neurons. We used immunohistochemistry to label dystrophin in cerebellar slices including the cerebellar cortex and CbN. As expected, we observed high dystrophin expression in wild-type PCs but little or no expression in *mdx* cells (WT: 55.6 ± 2.73 *n* = 21; *mdx* −4.7 ± 2.5, *n* = 18, *p* < 0.00001, Figure 5A1,B1,C). The remaining dystrophin labeling sometimes evident in *mdx* PCs likely results from the expression of shorter dystrophin isoforms also recognized by the MANDRA1 antibody, but not impacted by the truncating point mutation in *mdx* mice. Dystrophin labeling in wild-type YFP+ CbN cells was low; reduced ~10-fold compared to PCs (CbN: 6.91 ± 1.79, *n* = 29, *n* = 21, *p* < 0.00001, Figures 5Ai–5Aiii,C). Further, the low dystrophin labeling observed in wild-type CbN neurons was not reduced in *mdx* (8.65 ± 1.66, *n* = 42, *p* = 0.48, Figures 5Bi–Biii,C), suggesting little or no expression of full-length dystrophin in glutamatergic CbN neurons, consistent with a previous study (Stay et al., 2019). The labeling of short dystrophin isoforms surrounding blood vessels in the CbN was not different across genotypes (*p* = 0.46, Figures 5Ai,Bi, asterisk; Figure 5D), suggesting that dystrophin labeling efficiency was similar across samples. Using qRT-PCR, we also measured dystrophin mRNA expression in PCs, YFP+ CbN neurons, and cerebellar granule cells (GCs) in wild-type mice. To measure the total dystrophin expression (including long and short isoforms), we used primers directed to the common C-terminal domain, while primers directed to the N-terminal domain were used to selectively measure full-length (Dp427) dystrophin expression. Our mRNA results confirm what we observed at the protein level; while full-length dystrophin was expressed in PC (4.28 ± 0.74, *n* = 14, Figure 5E), little to no full-length mRNA was detected in glutamatergic CbN neurons (0.50 ± 0.27, *n* = 13, *p* = 0.00004, Figure 5E) or GC (0.1 ± 0.09, *n* = 8, *p* = 0.0003, Figure 5E). Interestingly, our data do suggest that shorter dystrophin isoforms may be expressed in glutamatergic CbN neurons (Figure 5F). These data show that the Dp427 isoform of dystrophin is highly expressed in PC but not in glutamatergic neurons of the CbN. This suggests that structural and functional changes observed at PC-CbN synapses in *mdx* mice are likely due to the loss of dystrophin from the presynaptic PCs.

### Loss of Dystrophin Alters Firing in Glutamatergic Projection Neurons

**Figure 6:**
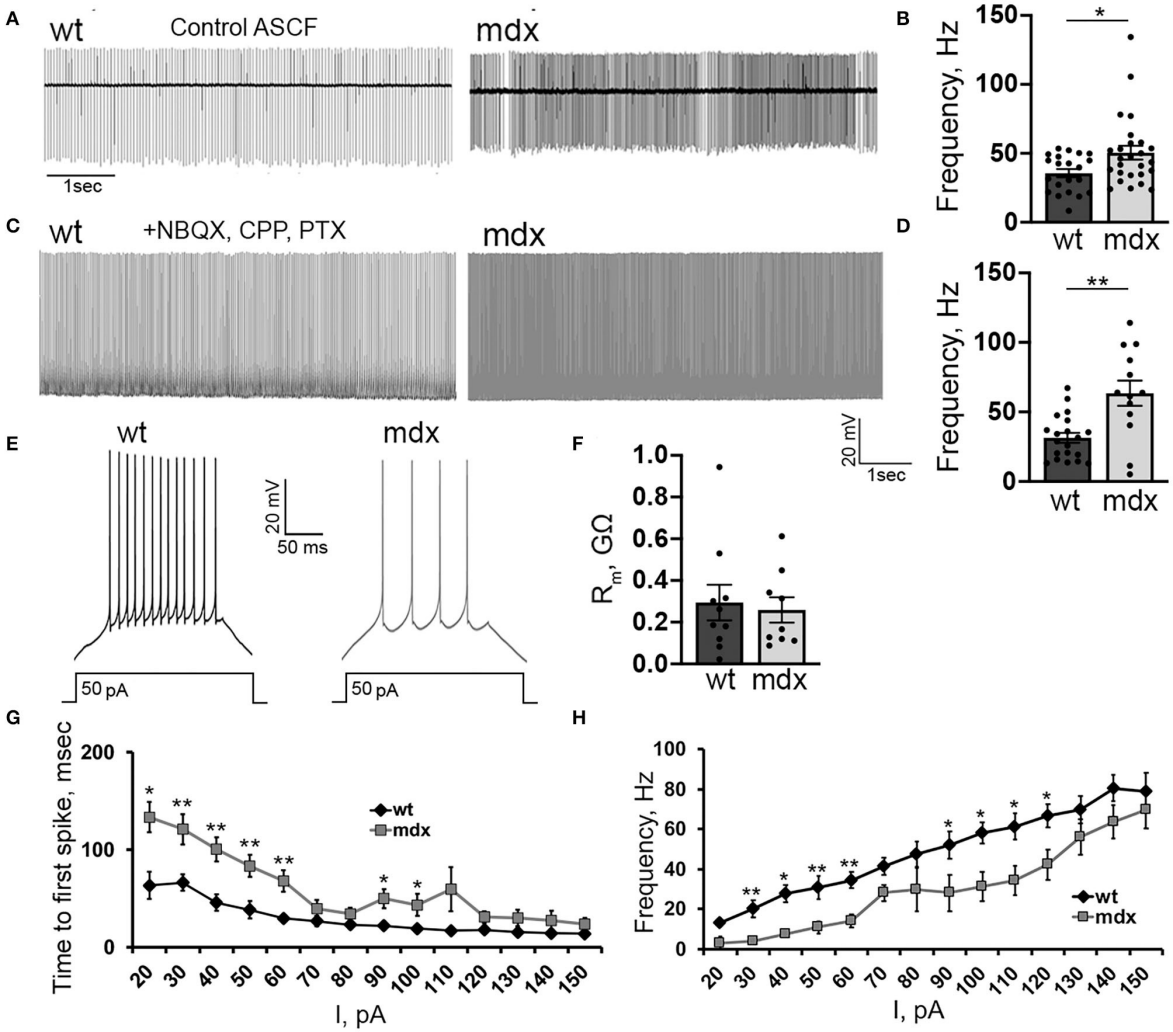
Loss of dystrophin alters the intrinsic excitability of CbN glutamatergic projection neurons. Representative traces **(A)** and mean firing rate **(B)** from cell attached recordings of spontaneous firing of wild-type (*n* = 11 cells from 3 animals) and *mdx* (*n* = 19 cells from 5 animals) YFP+ CbN neurons. Scale bar: 1 s. Representative traces **(C)** and mean firing rate **(D)** from whole-cell recordings of spontaneous action potentials recorded in the presence of NBQX, CPP, and PTX from YFP+ CbN neurons in wild-type (*n* = 19 cells from 5 animals) and *mdx* (*n* = 13 cells from 6 animals) mice. Scale bar: 20 mV, 1 s. **(E)** Representative traces of AP trains in current-clamped wild-type and *mdx* YFP+ CbN neurons evoked by 200 ms and 50 pA current injections. Scale bar: 20 mV, 50 ms. **(F–H)** Summary graphs of mean membrane resistance **(F)**, mean time to first action potential **(G)**, and mean firing frequency during depolarizing current injections **(H)** in wild-type (dark gray, *n* = 9 cells from 3 animals) and *mdx* (light gray, *n* = 8 cells from 3 animals) YFP+ CbN neurons. Error bars indicate SEM. *p ≤ 0.05, **p ≤ 0.01.

We also investigated firing properties of glutamatergic CbN neurons, which represent the primary output from the cerebellar circuit. In cell-attached recordings, we found that the spontaneous firing rate of YFP+ CbN neurons was faster in *mdx* mice (WT: 35.6 ± 2.9, *n* = 21; *mdx*: 50.5 ± 4.9, *n* = 26, *p* = 0.02, Figures 6A,B), possibly due to reduced inhibitory PC input. However, when fast synaptic transmission was blocked by the inclusion of NBQX, CPP, and PTX in the bath, we observed an even greater increase in spontaneous firing in *mdx* (WT: 31.4 ± 3.6, *n* = 20; *mdx*: 63.5 ± 9.02, *n* = 13, *p* = 0.00065, Figures 6C,D), suggesting that the difference in firing is due to changes in the intrinsic firing properties of these cells. To test this, we hyperpolarized CbN neurons to −65 mV (to silence spontaneous firing) and evoked AP firing through 200 ms depolarizing current injections (20–150 pA; in the presence of synaptic blockers). Surprisingly, we found that evoked firing was reduced in *mdx* neurons (Figure 6E). This was evident by reduced firing rates (50 pA current injection: WT: 30.6 ± 4.06 Hz, *n* = 9; *mdx*: 10.9 ± 3.2 Hz, *n* = 7, *p* = 0.003, Figure 6H) and increased latency to the first AP (50 pA current injection: WT: 38.4 ± 8.9 ms, *n* = 9; *mdx* 83.5 ± 9.6 ms, *n* = 7, *p* = 0.01, Figure 6G) during each current injection. These differences are not accounted for by changes in input resistance, which did not differ between genotypes (*p* = 0.74, Figure 6F). Instead, this raises the possibility that active currents are altered in *mdx* CbN neurons.

**Figure 7:**
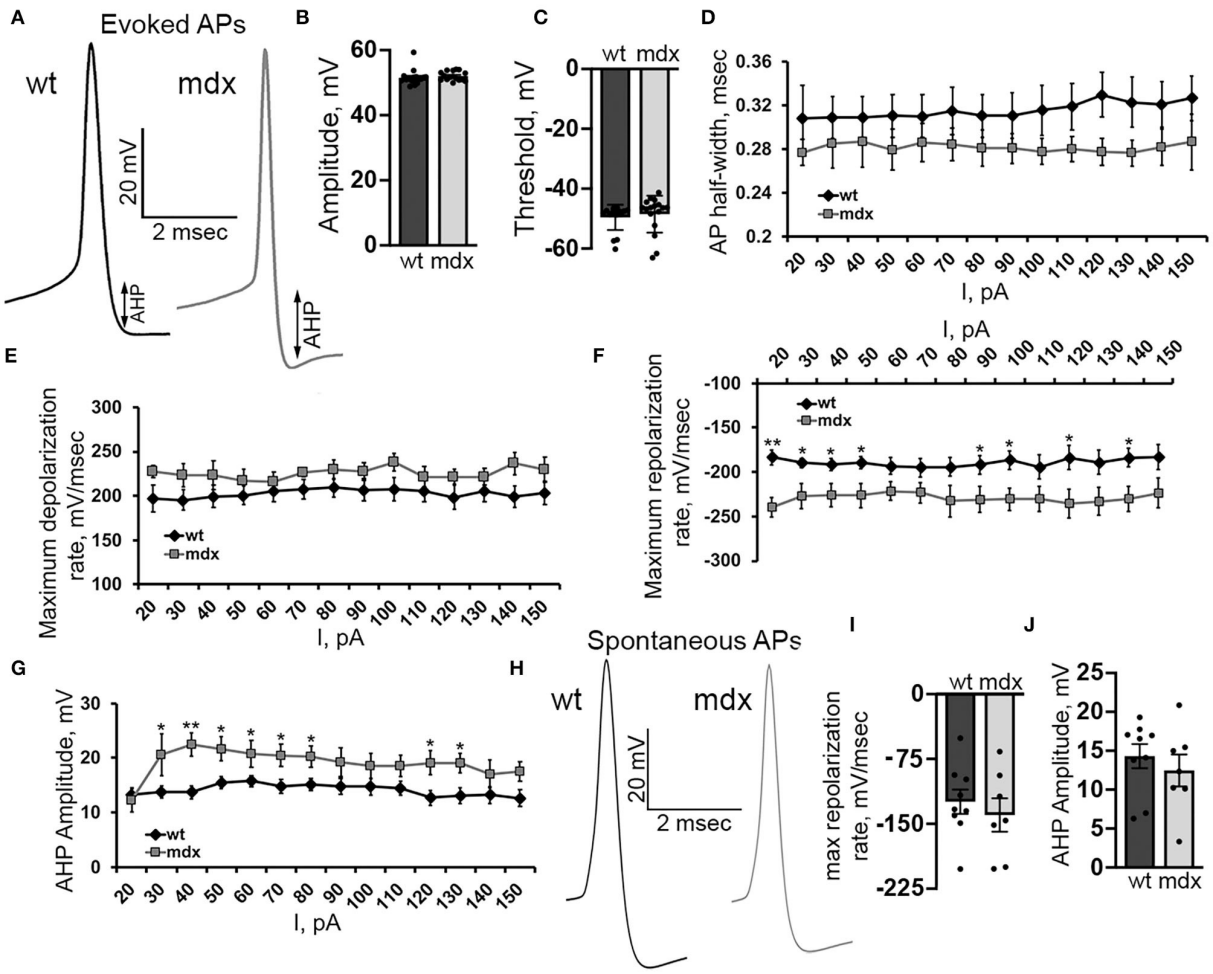
Evoked AP repolarization rate and AHP are increased in *mdx* CbN neurons. **(A)** Representative traces of evoked AP waveforms from wild-type (black trace) and *mdx* (gray trace) YFP+ CbN neurons. Arrows indicate AHP amplitude. Scale bar: 20 mV, 2 ms. **(B,C)** Summary graphs of mean AP amplitude **(B)** and threshold **(C)** from current clamped wild-type (dark gray bar, *n* = 9 cells from 3 animals) and *mdx* (light gray bars, *n* = 8 cells from 3 animals) YFP+ CbN neurons. **(D–G)** Summary graphs of mean AP half width **(D)**, maximum depolarization rate **(E)**, maximum repolarization rate **(F)**, and mean AHP amplitude **(G)** plotted against current injection from wild-type (black diamonds, *n* = 9 cells from 3 animals) and *mdx* (gray squares, *n* = 8 cells from 3 animals) neurons. **(H)** Representative traces of spontaneous APs (no current injection through the patch pipette) recorded from wild-type (left, black trace) and *mdx* (right, gray trace) YFP+ CbN neurons. Traces are averages of 200 action potentials. Scale bar: 20 mV, 2 ms. **(I,J)** Summary graphs of mean maximum repolarization rate **(I)** and AHP amplitude **(J)** of spontaneous APs in wild-type (dark gray bars, *n* = 9 cells from 2 animals), and *mdx* (light gray bars, *n* = 8 cells from 3 animals) neurons. Error bars indicate SEM. *p ≤ 0.05, **p ≤ 0.01.

In order to better understand changes in evoked firing, we examined the AP waveform in wild-type and *mdx* CbN neurons (Figure 7A). The AP amplitude, threshold, and slope of the raising phase were not different between genotypes [50 pA current injection *n* = 18 (WT), 17 (*mdx*), *p* = 0.35–0.55, Figures 7B,C,E]. However, we did observe a faster repolarization of the AP, measured by the maximum negative slope during the repolarizing phase of the AP (50 pA current injection: WT: 189.7 ± 7.0 mv/ms, *mdx*: 226.5 ± 13.6 mv/ms, *p* = 0.02, Figure 7F), a larger AHP (50 pA current injection: WT: 15.5 ± 1.2 mV, *mdx*: 21.6 ± 2.2 mV, *p* = 0.02, Figure 7G), and a trend toward smaller AP half-width (50 pA current injection: WT: 0.31 ± 0.02 ms; *mdx*: 0.28 ± 0.02 ms, *p* = 0.32, Figure 7D). Together, these data suggest a greater voltage- or calcium- dependent K^+^ current activated during evoked AP discharge in CbN neurons of *mdx* mice. Interestingly, we did not observe any differences in the waveform of spontaneous AP in wild-type and *mdx* neurons (max repolarization rate *p* = 0.52, AHP amplitude *p* = 0.48 Figures 7H–J). This suggests that the larger putative K^+^ current observed in *mdx* neurons may be inactivated at the more depolarized membrane potentials present during spontaneous firing. This may explain at least in part, why spontaneous firing is increased in *mdx* neurons, but evoked firing is reduced. Together these observations suggest that the loss of dystrophin in PCs causes downstream changes in glutamatergic projection neurons of the CbN altering both spontaneous and evoked firing.

## Discussion

Several lines of evidence suggest altered cerebellar function may contribute to DMD pathologies (Cyrulnik and Hinton, 2008). However, previous cellular studies have mainly focused on PCs in the cerebellar cortex. Here, we examine synaptic and firing changes in glutamatergic projection neurons of the CbN, which form the primary output of the cerebellar circuit and project to numerous regions of the CNS. We find that inhibitory synaptic drive from PCs, the primary input to CbN neurons, is significantly reduced in *mdx* mice. Furthermore, we find that spontaneous AP firing is increased, but evoked firing from a hyperpolarized baseline is reduced, suggesting changes in the intrinsic excitability of these neurons. These data, together with previous studies of PC activity in *mdx* mice (Anderson et al., 2003; Kueh et al., 2008; Wu et al., 2022), suggest that information processing through the cerebellar circuit and the output of cerebellar neurons may be impaired by the loss of dystrophin expression, potentially contributing to motor and cognitive deficits observed in DMD.

This work employed the *mdx* mouse line, which lacks dystrophin (Dp427) expression in all tissues. Using a global null mouse model is beneficial because it may more closely recapitulate changes in the cerebellar circuit present in the human disease. However, from a mechanistic point of view, a global null makes it difficult to correlate specific phenotypes with loss of dystrophin from specific cell types. Given the high expression of dystrophin in PCs, and lack of expression observed in CbN neurons (Figure 5, Stay et al., 2019), it is likely that changes in the cerebellar output observed here result from the loss of dystrophin normally expressed in PCs. However, we cannot rule out effects due to changes in the cerebellar input, potentially produced by changes in CNS circuits elsewhere, or altered motor feedback from the periphery caused by impaired muscle function. A mechanistic understanding of changes in the cerebellar circuit will require cell-specific ablation of dystrophin using conditional knockout mice in future studies.

### Altered Inhibitory Synaptic Transmission in CbN Neurons

Glutamatergic projection neurons of the CbN receive excitatory input primarily from axon collaterals of mossy fibers and inhibitory input from PC axons (Chan-Palay, 1977). However, unlike many neurons, which are generally silent until receiving excitatory input, CbN neurons fire spontaneously at high rates and are silenced by elevated inhibition. CbN neurons receive hundreds to thousands of synaptic contacts from PCs, most of which target the soma and axon hillock (Chan-Palay, 1977), providing a powerful inhibitory drive to these cells that largely controls their firing behavior (Person and Raman, 2012). We found that the frequency of mIPSCs in CbN neurons is significantly reduced. This finding, coupled with a lack of change in the PPR or P_r_ value measured from cumulative IPSC analysis, suggests that the number of functional PC synapses is reduced. This finding is further supported by a reduction in the number of anatomically defined synapses on the soma of CbN neurons using immunohistochemistry. This result is similar to recent findings in PCs, where functionally and anatomically defined inhibitory synapses are also reduced (Wu et al., 2022). However, in PCs, dystrophin and the dystrophin-associated glycoprotein complex are clearly localized to inhibitory postsynaptic densities (Knuesel et al., 1999), where they potentially bind to presynaptic neurexin proteins (Sugita et al., 2001) and act as synaptic adhesion molecules (Briatore et al., 2020; Wu et al., 2022). We did not observe clear dystrophin protein or mRNA expression in CbN neurons using immunohistochemistry or single cell qRT-PCR, suggesting that dystrophin is not normally expressed postsynaptically and the loss of inhibitory synapses in these cells likely proceeds through a different mechanism, possibly as compensation for firing changes in upstream PCs (Stay et al., 2019; Wu et al., 2022).

We also observed increased short-term depression and slower recovery from depression during high-frequency stimulation at inhibitory synapses in *mdx* CbN neurons. Desensitization of postsynaptic GABA_A_ receptors is limited at PC synapses in the CbN (Pugh and Raman, 2005), suggesting increased short-term depression involves a presynaptic mechanism. This could indicate expression of dystrophin in PC terminals, as has been observed at photoreceptor terminals of the retina (Schmitz and Drenckhahn, 1997). However, we did not observe elevated dystrophin labeling surrounding the soma of CbN neurons, where dense PC terminals are observed. Though it should be noted that background fluorescence was high in the CbN region, and it is possible that low levels of presynaptic dystrophin expression were not detected. It is also possible that the reduced vesicle replenishment is compensation for changes in PC firing. *In vivo* recordings of PCs in awake *mdx* mice show little change in spontaneous firing compared to wild-type cells, but greater positive-modulation of PC firing (i.e., greater high-frequency bursting) in response to an unexpected sensory stimulus (Wu et al., 2022). Reduced fast vesicle replenishment at PC-CbN synapses attenuates high-frequency bursts of activity (due to greater short-term depression) while having little effect on transmission at lower firing rates, overall, reducing the dynamic range of signaling at PC-CbN synapses. In addition to altering the firing behavior of CbN neurons, reduced high-frequency transmission at these synapses is also likely to disrupt the induction of synaptic plasticity at mossy fiber and PC synapses, which depend on elevated inhibition (Aizenman et al., 1998; Pugh and Raman, 2006).

### Altered Excitability of Cerebellar Nuclear Neurons

Like PCs, CbN neurons fire spontaneous APs in the absence of synaptic input. In cell-attached and whole-cell recordings from CbN neurons, we found that the spontaneous firing rate was significantly increased in *mdx* mice, suggesting increased excitability of these cells. Surprisingly, we also found that evoked firing from a hyperpolarized baseline was reduced in *mdx* neurons. This was evident in both a greater time to the first AP and reduced firing frequency throughout the depolarization. Analyzing the waveform of evoked APs, we found that the repolarization rate and AHP amplitude were greater in *mdx* cells, suggesting a larger voltage- or calcium-gated K^+^ current which could account for reduced evoked firing in these cells. Interestingly, we did not observe any differences in spontaneous AP waveforms across genotypes. These observations can be explained by an increased voltage- or calcium-gated K^+^ current in *mdx* CbN neurons that is active when firing is initiated from a hyperpolarized baseline, but largely absent (possibly due to inactivation) during spontaneous firing occurring from a relatively depolarized baseline (Raman et al., 2000). The net effect of firing changes in the CbN, increased spontaneous firing but reduced evoked firing, is likely to be a reduced dynamic range of firing in *mdx* CbN neurons, and therefore, reduced dynamic range of the output of the cerebellum itself. Overall, CbN neurons may be less sensitive to either excitatory inputs, due to reduced evoked firing, or inhibitory inputs, due to reduced inhibitory innervation and high-frequency transmission.

### Cerebellar Dysfunction Contributes to DMD

Several lines of evidence suggest that cerebellar impairment is involved in cognitive deficits (Cyrulnik and Hinton, 2008) and comorbidity with autism spectrum disorder (Fujino et al., 2018; Darmahkasih et al., 2020; Wu et al., 2022) in DMD. The cerebellum forms processing loops with several areas of cortex (*via* afferent projections through the pontine nuclei, and efferent projections to the thalamus), including motor, parietal, and prefrontal cortex (Middleton and Strick, 2001; Sultan et al., 2012). Reciprocal connections with prefrontal cortex in particular suggest that the cerebellum participates in decision making and complex social behavior. In fact, increasing evidence suggests that cerebellar dysfunction (such as occurs following cerebellar stroke) can produce a range of cognitive deficits (Schmahmann, 2019) and cerebellar deficits in early development may contribute to autism spectrum disorder (Wang et al., 2014). Previous studies have shown impaired PC function, including altered synaptic transmission, synaptic plasticity, and PC firing, in *mdx* mice (Anderson et al., 2003, 2004; Stay et al., 2019; Wu et al., 2022). Our work shows that output neurons of the cerebellar circuit are also impaired by global loss of dystrophin. Specifically, we find that the dynamic range of PC inputs and CbN firing are reduced in *mdx* mice. Together, these data suggest that associative learning, information processing, and output firing in the cerebellar circuit may be impaired due to the loss of dystrophin expression. Altered feedback from the cerebellum during early development may alter cortical development (Wang et al., 2014) and explain the high association between DMD and neurodevelopmental disorders, such as autism spectrum disorder. Further, altered information processing in the cerebellar circuit and reduced dynamic range in the cerebellar feedback to prefrontal cortex and hippocampus likely contributes to cognitive deficits and reduced verbal skills observed in *mdx* mice and humans with DMD.

## Data Availability Statement

The raw data supporting the conclusions of this article will be made available by the authors, without undue reservation.

## Ethics Statement

The animal study was reviewed and approved by Institutional Animal Care and Use Committee at University of Texas Health at San Antonio.

## Author Contributions

TK-P and JP designed the experiments, wrote, and edited the manuscript. TK-P collected and analyzed all the data. All authors contributed to the article and approved the submitted version.

## Funding

This work was supported by the U.S. National Institutes of Health Grant NS123933 (JP) and a Young Investigator Award (JP) from the Max and Minnie Tomerlin Voelcker Fund.

## Conflict of Interest

The authors declare that the research was conducted in the absence of any commercial or financial relationships that could be construed as a potential conflict of interest.

## Publisher's Note

All claims expressed in this article are solely those of the authors and do not necessarily represent those of their affiliated organizations, or those of the publisher, the editors and the reviewers. Any product that may be evaluated in this article, or claim that may be made by its manufacturer, is not guaranteed or endorsed by the publisher.

## Supplementary Material

Supplementary Figure 1 **(A)** DIC image (left) and fluorescence image (using illumination from a 473 nm LED and a GFP filter set, right) of CbN neurons showing strong YFP expression in a subset of CbN neurons in the Thy1-YFP16 mouse line. ^*^YFP- (non-glutamatergic) neurons, ^**^YFP+ (glutamatergic) neuron. **(B)** Graph showing mean mRNA expression (normalized to actin) of VGlut2 (glutamatergic neuron marker) in PCs (dark gray bar, *n* = 12 cells from 2 animals) and YFP+ CbN neurons (light gray bars, *n* = 14 cells from 2 animals) using single cell patch qRT-PCR. Error bars indicate SEM. ^*^*p* ≤ 0.05.
