# Supplementary figures and images for: Altered Synaptic Transmission and Excitability of Cerebellar Nuclear Neurons in a Mouse Model of Duchenne Muscular Dystrophy
Source: Front Cell Neurosci. 2022 Jul 5;16:926518. doi: 10.3389/fncel.2022.926518 (PMC9294606; doi:10.3389/fncel.2022.926518)

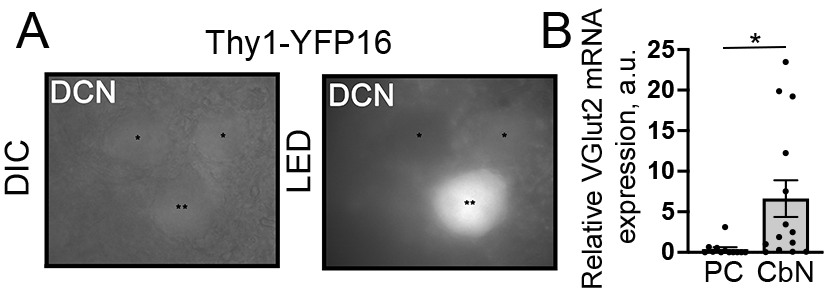

Supplement: Supplementary Figure 1 — (A) DIC image (left) and fluorescence image (using illumination from a 473 nm LED and a GFP filter set, right) of CbN neurons showing strong YFP expression in a subset of CbN neurons in the Thy1-YFP16 mouse line. *YFP- (non-glutamatergic) neurons, **YFP+ (glutamatergic) neuron. (B) Graph showing mean mRNA expression (normalized to actin) of VGlut2 (glutamatergic neuron marker) in PCs (dark gray bar, n = 12 cells from 2 animals) and YFP+ CbN neurons (light gray bars, n = 14 cells from 2 animals) using single cell patch qRT-PCR. Error bars indicate SEM. *p ≤ 0.05. [file Image_1.TIF]
